# Predicting the quality attributes related to geographical growing regions in red-fleshed kiwifruit by data fusion of electronic nose and computer vision systems

**DOI:** 10.1186/s12870-023-04661-6

**Published:** 2024-01-02

**Authors:** Mojdeh Asadi, Mahmood Ghasemnezhad, Adel Bakhshipour, Jamal-Ali Olfati, Mohammad Hossein Mirjalili

**Affiliations:** 1 Department of Horticultural Sciences, Faculty of Agricultural Sciences, University of Guilan, Rasht, Iran; 2 Department of Biosystems Engineering, Faculty of Agricultural Sciences, University of Guilan, Rasht, Iran; 3 Department of Agriculture, Medicinal Plants and Drugs Research Institute, Shahid Beheshti University, Tehran, Iran

**Keywords:** Image processing, Machine learning, Origin, Physicochemical attributes, Volatile Organic compounds

## Abstract

The ability of a data fusion system composed of a computer vision system (CVS) and an electronic nose (e-nose) was evaluated to predict key physiochemical attributes and distinguish red-fleshed kiwifruit produced in three distinct regions in northern Iran. Color and morphological features from whole and middle-cut kiwifruits, along with the maximum responses of the 13 metal oxide semiconductor (MOS) sensors of an e-nose system, were used as inputs to the data fusion system. Principal component analysis (PCA) revealed that the first two principal components (PCs) extracted from the e-nose features could effectively differentiate kiwifruit samples from different regions. The PCA-SVM algorithm achieved a 93.33% classification rate for kiwifruits from three regions based on data from individual e-nose and CVS. Data fusion increased the classification rate of the SVM model to 100% and improved the performance of Support Vector Regression (SVR) for predicting physiochemical indices of kiwifruits compared to individual systems. The data fusion-based PCA-SVR models achieved validation R^2^ values ranging from 90.17% for the Brix-Acid Ratio (BAR) to 98.57% for pH prediction. These results demonstrate the high potential of fusing artificial visual and olfactory systems for quality monitoring and identifying the geographical growing regions of kiwifruits.

## Introduction

Kiwifruit (*Actinidia chinensis*) is a significant fruit crop, both economically and nutritionally. It originated in China but is now cultivated worldwide [1]. In recent years, the demand for red kiwifruit has increased among consumers due to its unique flavor and brightly colored pericarp [2].

The nutritional value of fruits and vegetables is influenced by several factors, including the location of the orchard, environmental conditions, cultivation techniques, and pedoclimatic aspects. Climatic conditions in different regions affect the composition of agricultural products, resulting in variations in protein, vitamin, sugar, acid, mineral, and aroma compound content, which in turn lead to differences in quality [3–6]. Guilan was the first province in Iran to produce kiwifruit, due to its mild and subtropical climate, which is suitable for cultivating various kiwifruit cultivars. The main red-fleshed variety grown in Iran is called 'ʿKhoni', characterized by its deep red core and a transverse section that reveals a striking combination of red and yellow-green colors.

Machine learning advancements allow for objective and accurate identification through quick and convenient processes. Electronic nose (E-nose) and computer vision systems (CVS) mimic the human olfactory and vision systems, respectively, to measure sample appearance and aroma [7]. CVS is a cross-disciplinary field focused on developing algorithms and methodologies to extract meaningful features from images of objects. This technique enables computers to perceive and understand crucial details about tangible objects from images [8]. The essence of CVS is comprised of image analysis and processing [9]. Image processing improves image quality by removing noise, while image analysis extracts numerical information by distinguishing objects from their surrounding [10]. In the food sector, CVS is used for examination and evaluation because it offers rapid, objective, consistent, and cost-effective assessment without damaging the product [11]. The successful application of CVS has been reported in various recent studies on fruits and vegetables [12–17].

The fragrance of kiwifruit is a crucial characteristic of its quality and a significant sensory property [18]. As living organisms, fruits, including kiwifruit, release volatile organic compounds (VOCs) that are influenced by environmental factors [19]. The composition of emitted VOCs provides valuable information about the fruit's health and freshness [20]. E-nose is a type of artificial odor-sensing equipment that includes a set of semi-selective gas sensors designed to mimic the human sense of smell [21, 22]. E-nose is a device that consists of a sensor group and data processing tools that detect different volatile compounds present in a sample's headspace, creating a unique "fingerprint" of the sample's compounds [23, 24]. There are several types of e-noses, including those that use metal oxide semiconductor sensors, thermal sensors, piezoelectric sensors, or mass spectrometers [18].

Volatile compounds, which are significant indicators of food quality, are found to be related to the origin of cultivation [25]. The application of e-nose technology has been successfully applied to identify the origin of various products, such as camellia seed oils [26], coffee [27], orange [28], and grape [29].

Data fusion is a technique that combines data from several resources to generate an integrated data vector, producing more accurate, consistent, and concise information than any individual data source. Generally, the integration of fusion has different benefits, including enhanced data authenticity (improved detection, confidence, and reliability, reduced data ambiguity) and data availability (extended spatial and temporal coverage) [30]. By combining a suitable algorithm to analyze the image obtained from CVS and the distinctive "fingerprint" gathered from the e-nose, valuable information about the samples can be extracted for identification purposes. The detection process using e-nose and CVS techniques does not require complex sample pre-treatments. Additionally, CVS detection for a single sample takes only a few seconds, while e-nose detection time can be reduced to a short time. This demonstrates that these two detection methods are both time-efficient.

Xu et al. [31] employed a data fusion approach based on e-nose and CVS to discriminate the geographical origin of Longjing teas with 100% accuracy. The data fusion strategy was also implemented using a combination of e-nose data and other emerging methods, such as electronic tongue [32] and hyperspectral imaging [33] for geographical origin assessment of agricultural products.

To the best of our knowledge, no research has been reported on the geographical origin and quality assessment of kiwifruit using both CVS and e-nose approaches. This study aimed to differentiate the quality of the ʻKhoniʼ kiwifruit cultivar according to geographical origin using e-nose and CVS systems combined with a synergetic data fusion strategy. To understand the capability of visual and aroma-extracted data, and machine learning algorithms to inspect the geographical growing region of kiwifruits, this paper proposes a rapid, simple, and objective identification method for identifying the quality and origin of red-fleshed kiwifruits. To achieve this objective, the research details are as follows: (1) To determine the effect of the growing region on the physiochemical attributes of red-fleshed kiwifruit. A comprehensive physiochemical analysis was conducted for this purpose. (2) To obtain the aroma and appearance-related data from CVS and e-nose methods for kiwifruit samples from various geographical regions. In addition to data extract from e-nose signals, image processing algorithms are also developed to extract distinctive features from the images of whole kiwifruits and their half-cut view. (3) To develop machine learning algorithms utilizing e-nose and CVS data for identifying the quality and growing region of red-fleshed kiwifruit samples. (4) To employ a feature-level data fusion strategy by combining CVS and e-nose data to obtain more accurate cognitive and predictive models.

## Materials and methods

### The trial site and kiwifruit samples

**Fig. 1:**
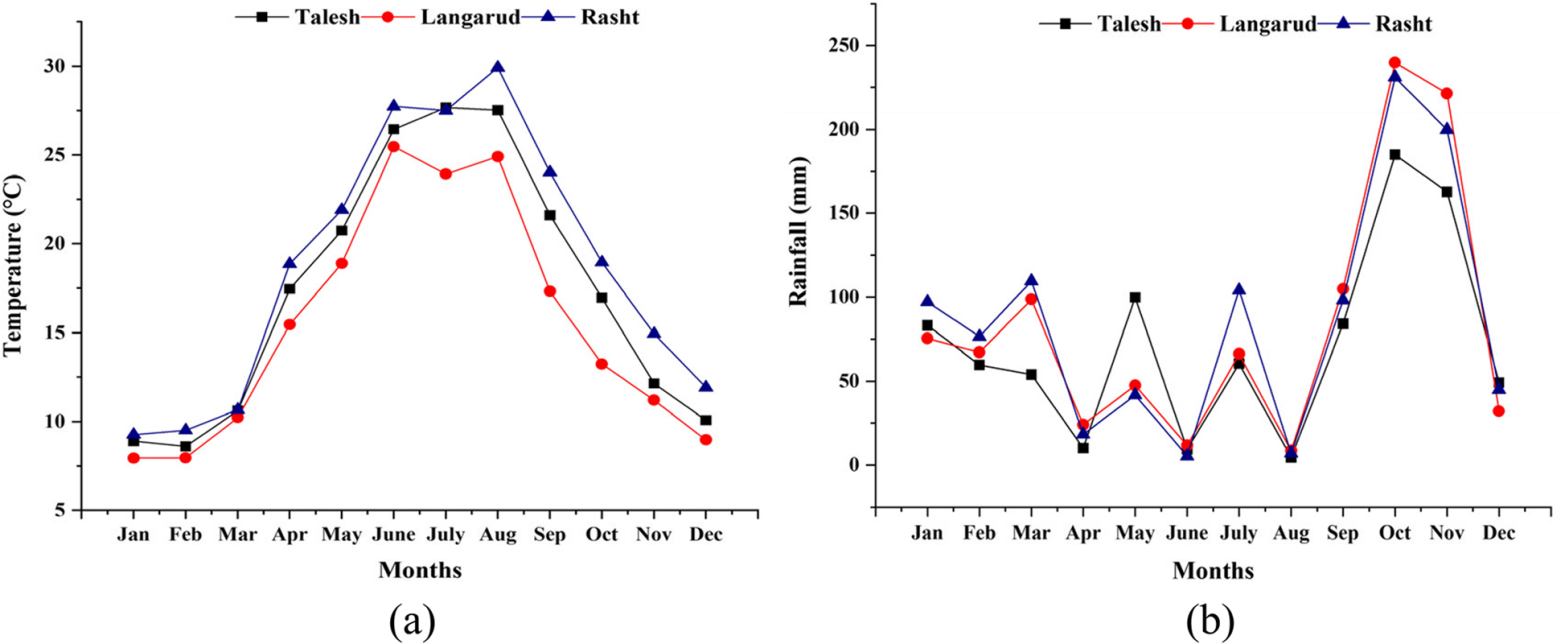
Climatic data of the three studied regions: **a** Temperature, **b** Rainfall [34]

This study investigated red-fleshed kiwifruit (*Actinidia chinensis* var. Khoni) from three different production regions in northern Iran, namely Talesh (west of Guilan), Langarud (east of Guilan), and Rasht (center of Guilan) (Table 1). The average monthly temperature and rainfall values for these three sites during the 2021 season are available in Fig. 1. Rasht had higher mean monthly temperatures than Talesh and Langarud. Langarud has colder weather in September and October, coinciding with the kiwifruit harvest season. Rasht also had the highest mean monthly rainfall.

**Table 1:** Location (region and coordinates) of three red kiwifruit groups selected for study

Orchard Region	Coordinates	Altitude (m)
Talesh	37.787231, 48.944723	6
Langarud	37.149168, 50.064294	121
Rasht	37.195375, 49.643989	27

The date of 90% female flower bud opening was recorded as April 21st for Rasht, April 24th for Talesh, and April 29th for Langarud. According to Choi et al. [35], all kiwifruits were harvested from uniform vines with the same irrigation, pruning, and fertilization practices, precisely 170 days after full bloom (DAFB). Immediately after harvest, the fruits were taken to the postharvest laboratory at the University of Guilan. The kiwifruit samples were selected based on their relatively uniform size and weight. The fruits were free of physical injuries, sunburn, blemishes, bruises, and pest and pathogen infestations. Upon arrival at the laboratory, the fruits were stored under controlled conditions at 20 °C with a relative humidity of 70% for seven days to facilitate natural ripening [35].

### Physicochemical determination

#### Firmness, Soluble Solids Content (SSC), pH, Titratable Acidity (TA), and Brix-Acid Ratio (BAR)

The firmness of fruits (kg/cm^2^) was measured using an Effegi penetrometer (model FT 011, USA) having a probe with an 8 mm diameter. The probe was placed at two locations on opposing sides of the equatorial plane of the fruit after peeling off a 2 mm layer from the outer surface of the fruit [36]. Kiwifruit juice was used to measure acidity and soluble solids content (SSC). The SSC value of the samples was determined as a percentage of sugars (%) using a digital pocket refractometer (Euromex RD 635, Netherlands).

Measurement of TA represented as the concentration of citric acid (%), involved titrating the fruit juice against 0.1 N NaOH while using phenolphthalein as the pH indicator [37]. To calculate the BAR, the SSC value was divided by the TA value. The pH of kiwifruit juice was measured by a digital pH meter (Hanna instrument, model HI 8519, Italy) [38].

#### Vitamin C, Total Phenolic (TP), Total Anthocyanin Content (TAC) and Antioxidant capacity

The vitamin C content was measured by the 2,6-dichloroindophenol titration method, following the CNS GB/T 6195–1986 protocol [39]. To prepare the extracts, 50 g of kiwifruit ground with liquid nitrogen was extracted with 200 mL of ethanol/acetone (7:3, v/v) for 1 h at 37 °C, according to the method described by Du et al. [39]. The extract was then filtered through Whatman No. 41 paper and rinsed with 50 mL of ethanol/acetone (7:3, v/v). The residue was extracted again using the same parameters. The combined filtrates were stored at 40 °C for subsequent TAC, TP, and antioxidant capacity measurements.

The amount of TP was determined using the Folin-Ciocalteu colorimetric method [39]. The output was presented as mg Gallic acid equivalents (GAE) per 100 g fresh weight (FW) (mg GAE/100 g FW). TAC was calculated using the pH differential technique [40]. The result was presented as mg cyanidin-3-glucoside (CGE) per 100 g FW (mg CGE/100 g FW). In this study, two different approaches were used to measure antioxidant capacity with slight modifications. The 2-diphenyl-1-picrylhydrazyl (DPPH) scavenging activity was measured with minor changes to the previously published method [41]. The fluorescence recovery after the photobleaching (FRAP) assay was conducted as reported by Du et al. [39] with some adjustments. The results were expressed as μM of ascorbic acid per 100 g FW.

### Measurement of free sugar content (fructose, glucose, and sucrose) using high-performance liquid chromatography-evaporative light scattering detector (HPLC-ELSD)

The HPLC method described by Shanmugavelan et al. [42] was used to analyze the sugar content. A Eurospher 100–5 NH2 column (5 μm, 250 mm × 4.6 mm, Knauer, Germany) was used on a Waters Alliance 2695 liquid chromatography system (Waters Corporation Milford, MA, USA) with an evaporative light scattering detector (Alltech ELSD 800). The mobile phase consisted of 5% water (A) and 95% acetonitrile (B), as used by Agblevor et al. [43]. The analysis was performed at a flow rate of 1.5 mL/min and a temperature of 20°C. One gram of frozen kiwifruit sample was blended with 10 mL of distilled water and centrifuged at 2000 rpm for 10 min using a freezing centrifuge (SIGMA 3-30 K, Heraeus Co., Germany). The resulting composition was filtered through a filter with 0.45 μm pore size. Free sugars, including fructose, glucose, and sucrose, were determined using an accurate and sensitive HPLC-ELSD method.

### Image acquisition

**Fig. 2:**
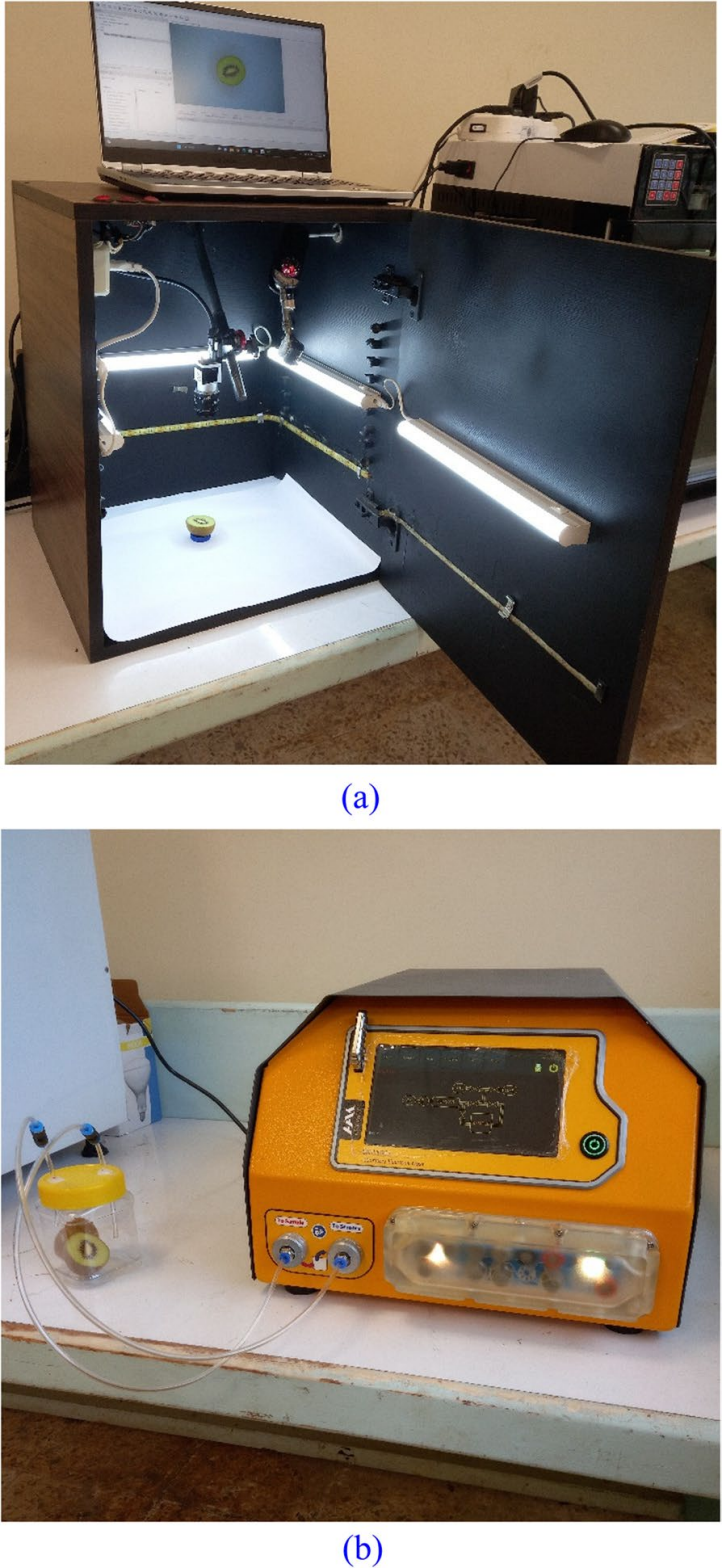
Actual views of the used computer vision (**a**) and e-nose (**b**) systems

To capture images of the kiwifruit samples, we used an apparatus developed by the Department of Biosystems Engineering of the University of Guilan (Fig. 2a). The main components of the image-capturing system were an illumination chamber with a lighting system and a 10-megapixel digital camera (Basler acA3800-10gc, Basler AG, Germany) with a user interface (Basler Pylon viewer application, Version 6.3.0.23157, Basler AG, Germany). The samples were placed on matte white cardboard in the illumination chamber, and the camera was positioned 20 cm above the samples. The camera was manually set to the same parameters for all image captures, and RGB images with a resolution of 3840 × 2748 pixels were captured and saved.

### Image processing and feature extraction

In this study, visual features were extracted from both whole and middle-cut images of kiwifruit samples.

To prepare the required images, a CVS was used, comprised of a wooden case with a matte black color, a lighting system, and a CCD camera. The details of this system were presented in a previous article [44]. The camera was mounted 20 cm above the samples, and color images with a resolution of 2736 × 3648 pixels were captured. To prepare images of the middle-cut surface of the samples, the kiwifruit samples were cut in the middle along the equatorial plane and placed inside the chamber on a ring with the cut surface facing the camera lens.

**Fig. 3:**
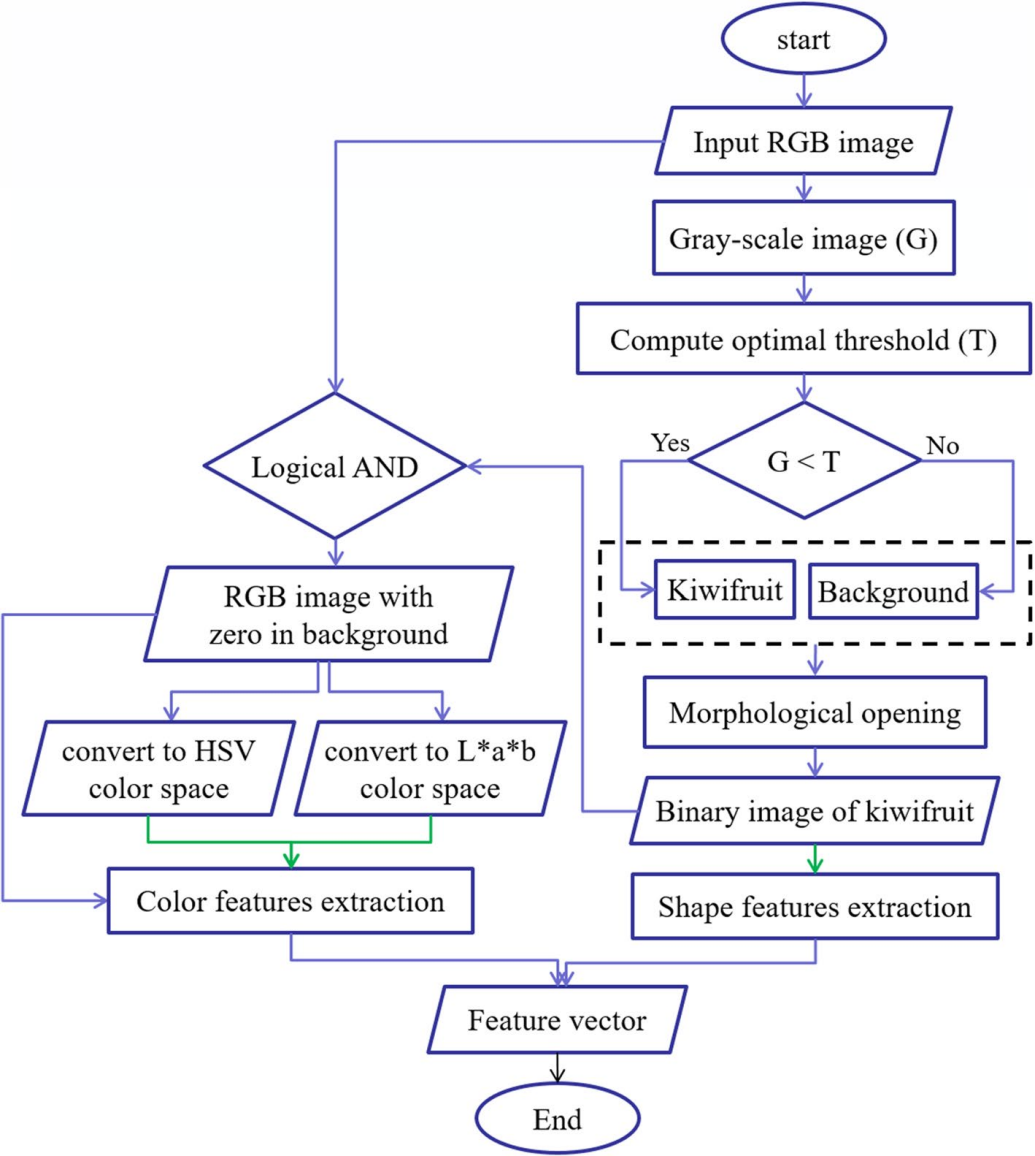
Flowchart of image processing and feature extraction from images of whole kiwifruits

**Fig. 4:**
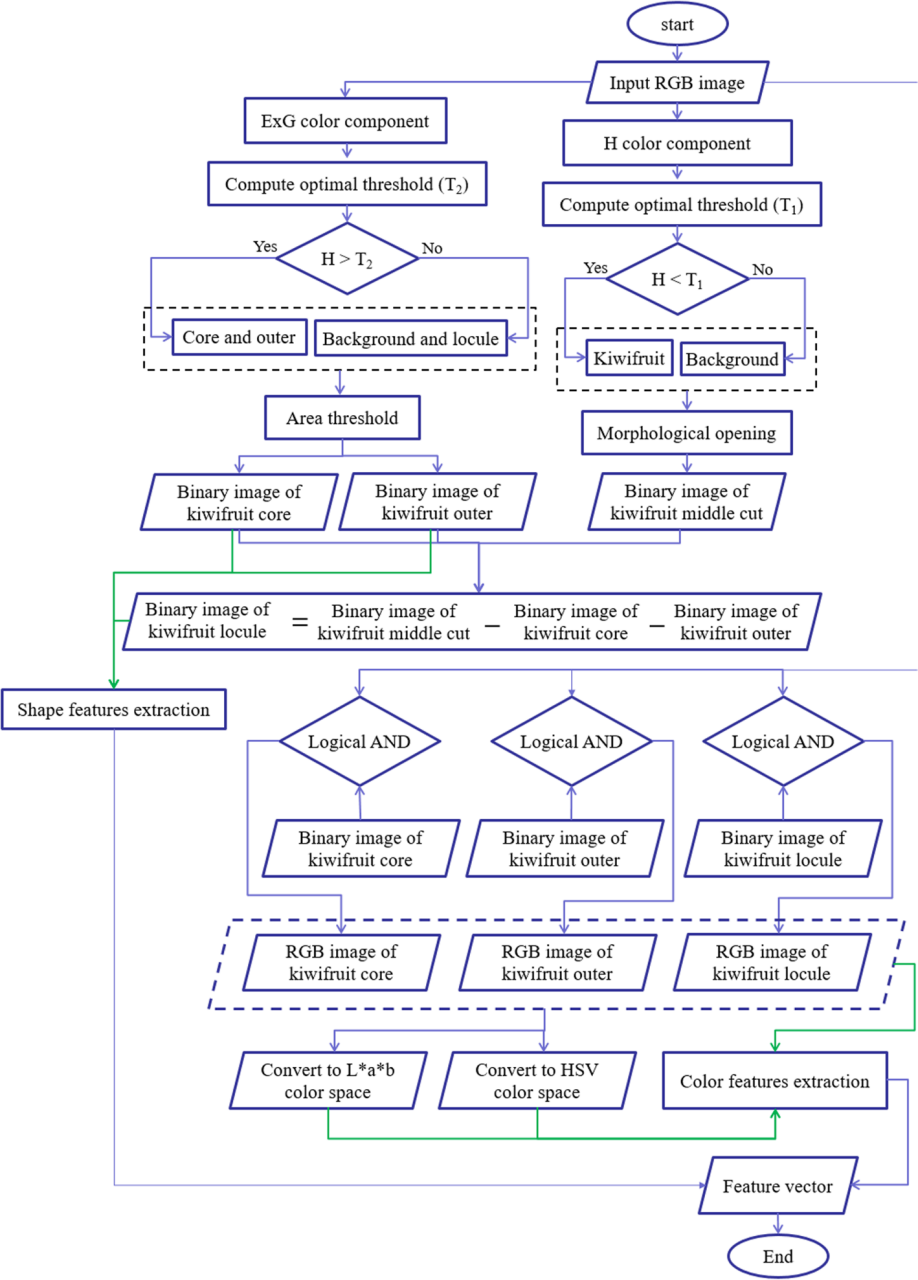
Flowchart of image processing and feature extraction from images of middle-cut section of kiwifruits

**Fig. 5:**
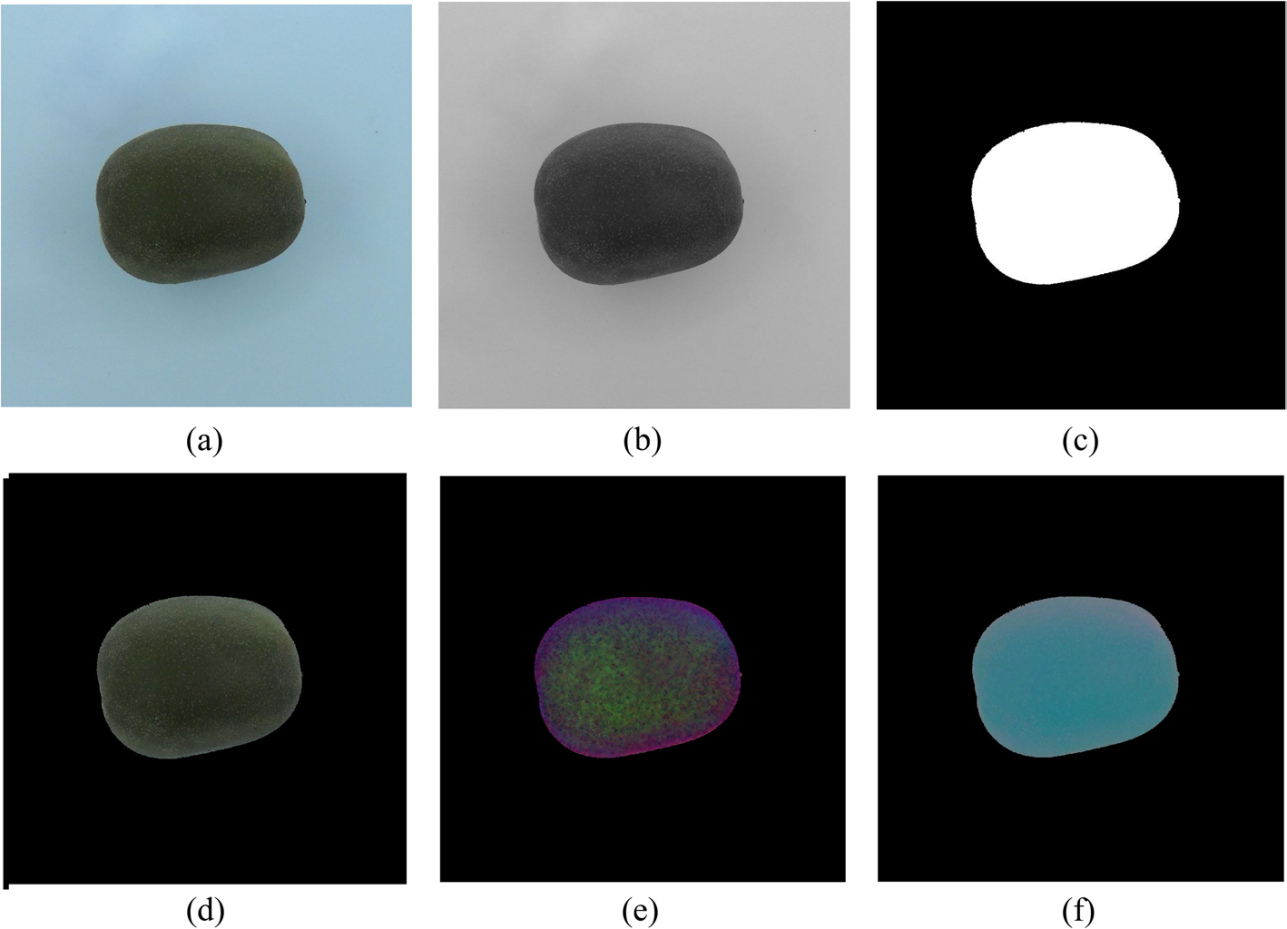
Gallery of whole fruit images at different steps of image processing; **a** RGB image of whole kiwifruit, **b** gray-scale image, **c** binary image of whole kiwifruit, **d** The whole kiwifruit RGB image resulted by applying logical AND between RGB image and binary image, **e** converted to HSV color space, and **f**) converted to L*a*b* color space

**Fig. 6:**
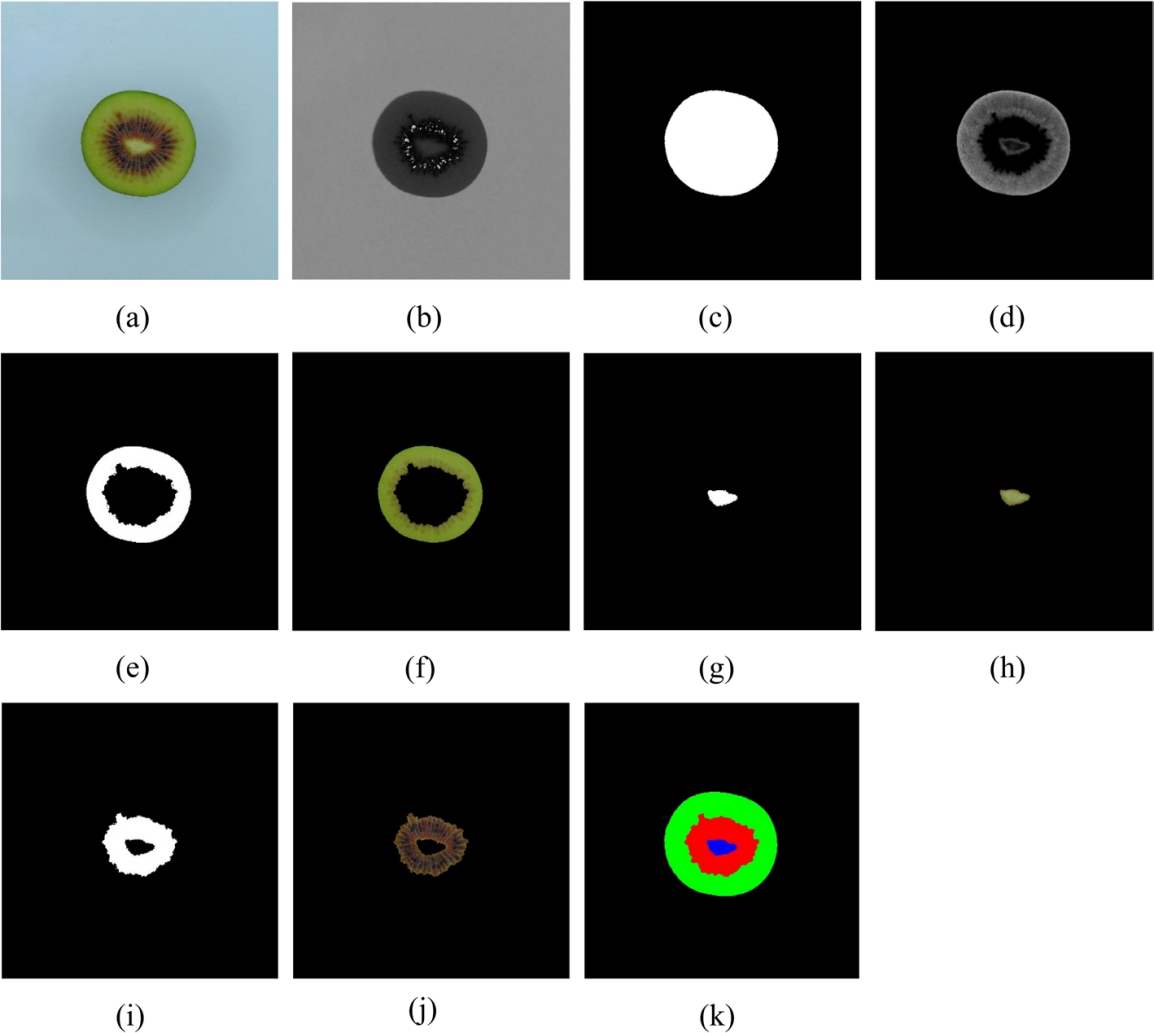
Gallery of middle-cut images at different steps of image processing; **a** RGB image of kiwifruit middle cut, **b** H component, **c** binary image of fruit middle-cut, **d** ExG image, **e** binary image of outer region, **f** RGB image of outer region, **g** binary image of core region, **h** RGB image of core region, **i** binary image of locule region, **j** RGB image of locule region, and **k**) colored image of segmented image

Table 2 summarizes the features extracted from whole kiwifruit and their middle cuts. To extract visual features, the RGB images of whole and middle-cut kiwifruit were transferred to the computer and analyzed in the image processing toolbox of MATLAB software (R2021a, the MathWorks, USA). Figures 3 and 4 show flowcharts of the image processing and feature extraction steps for whole and middle-cut images of kiwifruit, respectively. Because the samples were located in almost the center of the images, in order to reduce the computational operations, at the beginning of the analysis, a block of 1600 × 1600 pixels was cut around the image center (Fig. 5a, and 6a) and used in the next steps. The whole fruit images were converted to grayscale (Fig. 5b) and by applying an optimal threshold using Otsu method, were converted to binary images. The unwanted noises and gaps were removed using morphological opening operation (dilation followed by erosion) and the resulting binary image of the kiwifruit (Fig. 5c) was used to extract morphological features. The extracted morphological features were area, roundness, and aspect ratio. These features are commonly used and have been described in previous literature [45–47].

**Table 2:** The extracted visual features from different parts of kiwifruit samples

	Color features	Morphological features
Whole fruit	Averages of R, G, B, H, S, V, L, a*, and b* color components	Area, Roundness, aspect ratio
Middle cut surface	-	Area, Roundness, aspect ratio
Outer	Averages of R, G, B, H, S, V, L, a*, and b* color components	-
Locule	Averages of R, G, B, H, S, V, L, a*, and b* color components	Locule area rate
Core	Averages of R, G, B, H, S, V, L, a*, and b* color components	Area, Roundness, aspect ratio

Additionally, by applying a logical AND between the RGB image and binary image of the whole kiwifruit, a RGB image was obtained in which the background pixels were zero (Fig. 5d). This image was subsequently converted to HSV (Fig. 5e) and L*a*b* (Fig. 5f) color spaces and the corresponding average values of nine color components in these three spaces were calculated. These color spaces and color components are described by Sangwine and Horne [48].

In order to extract features from the kiwifruit middle-cut section (Fig. 6a), the following steps were performed: 1) Based on preliminary evaluations, the fruit section pixels were segmented by applying an optimal threshold to the hue color component (Fig. 6b). 2) The noises were removed using morphological opening operation and the binary image of the kiwifruit section was obtained (Fig. 6c). 3) To segment the outer and core parts, the excessive green value was calculated using ExG = 2*green–red-blue formula (Fig. 6d), which is also called excessive green [49]. 4) By optimal thresholding on the ExG image, the outer (Fig. 6e) and core (Fig. 6g) regions were segmented. 5) The locule region was obtained by subtracting the core and outer regions from the middle-cut binary image (Fig. 6i). 6) Morphological data were extracted from the binary images of the middle-cut section and core segment. Additionally, the ratio of the locule area to the middle cut surface area was calculated. 7) These binary images were overlaid with the original RGB image to obtain the RGB images of the outer, core, and locule regions (Fig. 6f, h, and j, respectively). 8) The color images of the outer, core, and locule regions were converted from RGB to HSV and L*a*b* spaces, and the color components in these three color spaces were calculated. Figure 6k shows a colored image of the image segmentation results for better visualization. In total, 46 image-based features were extracted and used to discriminate the kiwifruit growing region and predict its quality attributes.

### Data acquisition by E-nose

To capture the volatile organic compound (VOC) data of the samples, a laboratory e-nose device (EN-L16Ca, Hamedan Azmoon Kavoshgar, Iran) was used (Fig. 2b). The system consisted of airflow shut-off valves, a sample container, a sensor-containing chamber, an electronic board for managing data acquisition, and a graphical monitoring and control interface. The e-nose system contained 13 metal oxide semiconductor (MOS) gas sensors, including 8 MQ sensors (Hanwei Electronics Co., Ltd.) and 5 TGS sensors (Figaro Electronic Co., Ltd.). The specifications of the sensors are provided in articles by Haghbin et al. [50] and Mirhoseini-Moghaddam et al. [51].

Every single sample was enclosed in a bottle of 250 ml capacity and kept for 15 min before olfactory evaluation to saturate the bottle with fruit volatiles. The e-nose system operated in three steps: 120 s for baseline correction, 40 s for headspace injection, and 60 s for sensor recovery. During the initial step, clean ambient air was supplied to the sensor chamber until the sensors stabilized. Subsequently, the kiwifruit sample headspace aroma was introduced into the sensor array, causing changes in the output voltage of each sensor during the injection phase. The final phase involved circulating fresh ambient air into the sensor cavity to eliminate any remaining sample scent left in the sensor chamber. The sensor responses during the entire 220 s process were recorded and transmitted to the computer for further analysis. MATLAB programming software was used to preprocess and extract features from the acquired e-nose signals. Equation 1 was used for signal normalization [52] where the raw response baseline is represented by Xs(0), and the normalized and raw responses of the sensor at time t are denoted as Ys(t) and Xs(t), respectively. The preprocessed data from the headspace phase between 121–140 s were chosen for feature extraction in this study.

1 $$Ys(t)=\frac{Xs\left(t\right)-Xs(0)}{{\text{Xs}}(0)}$$

After normalization, the maximum sensor response (MSR), which is the most common e-nose-based feature [53–55], was extracted and used for classification and prediction purposes. In total, 13 e-nose features (derived from the 13 sensors) were extracted and utilized.

### Machine learning algorithms

Machine learning algorithms were developed in Unscrambler X software (version 10.4, CAMO ASA, Oslo, Norway). To extract patterns of the most influential image-extracted and e-nose features for the geographical discrimination of kiwifruits, Principal component analysis (PCA) was performed. PCA is a dimensionality reduction method that produces a set of new feature vectors without intercorrelations, named principal components (PCs), which are linear combinations of the primary features [56].

Because of the large number of input variables, the PCA-generated vectors were used for classification and prediction models instead of the primary calculated e-nose and CVS features. The first four PCs generated by PCA were fed into a support vector machine (SVM) classifier to discriminate kiwifruits based on their geographical growing region. These PC vectors were also used to predict the physicochemical attributes using support vector regression (SVR).

The SVM algorithm is a supervised machine learning method for either classification or regression problems. By creating an optimal hyperplane, the SVM algorithm separates the data into opposite groups with the maximum possible margin [57]. SVR is a modification to solve regression problems [58]. SVM and SVR models with different 2D polynomial kernels, cost values (0.01, 0.1, 1, 10, and 100), and gamma values (0.01, 0.1, 1, 10, and 100) were evaluated. The optimal parameters were selected using the grid sampling method and the smallest root mean squared error (RMSE) value of tenfold cross-validation. Data was randomly divided into calibration (two-thirds of data) and testing (one-third of data) datasets, and tenfold cross-validation was used in the calibration stage.

### Model evaluation

The best models for the prediction of physicochemical indices were selected based on the value of RMSE and coefficient of determination (R^2^) in the cross-validation stage. The models with the greatest R^2^ and the lowermost RMSE values were the desired ones. RMSE is a highly recommended performance criterion when the model outputs are numeric [59]. Another performance metric that was calculated for SVM classifiers was classification accuracy. The classification accuracy is calculated by dividing the number of correctly classified cases by the total number of instances. The descriptions and calculation formulas for these statistical indices are provided in previous literature [60, 61].

### Statistical analysis of quality attributes

Statistical analysis of the quality data was carried out using one-way ANOVA with SAS software (version 9.4, SAS Institute Inc., Cary, NC, USA) through Tukey’s HSD test at *p*-value < 0.05 significance level.

## Results and discussion

### Results of physicochemical indices

The results of the analysis of variance (ANOVA) in Table 3 show that the growing location had a highly significant effect on firmness, SSC, BAR, pH, vitamin C, TP, DPPH, FRAP, and TAC (all *p* < 0.01). Meanwhile, the TA was not significantly influenced by the growing location (*p* < 0.05). The fruits from Langarud (east of Guilan) had the highest SSC (20.96°Brix), SSC/TA (38.12), vitamin C (94.19 mg/100 g), TP (104.91 mg GAE/100 g), DPPH (67.12%), FRAP (10.68 μmol ascorbic acid/g) and TAC (3.49 mg CGE/100 g). The fruits grown in Rasht (center of Guilan) were firmer (1.20 kg/cm2) than those from Talesh and Langarud. The kiwifruits grown in Talesh (west of Guilan) had the highest pH (3.83), while the fruits from Rasht had the lowest. The significance of orchard and region was evident, given previous reports on variation between orchards [62, 63]. Previous literature found that the variation between orchards was related to differences in altitude and temperature. Environmental factors, such as light, temperature, and soil conditions, regulate anthocyanin pigmentation. Color intensity varies with altitude. The low temperature and high precipitation in Langarud may have caused the increase of TAC in kiwifruit in this region. Man et al. [62] reported that the decrease in anthocyanin biosynthetic gene expression in higher temperatures decreases anthocyanin concentration in red-fleshed kiwifruit.

**Table 3:** Variation of taste-related compounds, nutritional value and free sugars content in red kiwifruit according to region

**Location**	Firmness (kg/cm^2^)	SSC (°Brix)	TA (%)	BAR	pH	Vitamin C (mg/100 g)	TP (mg GAE/100 g)	DPPH (%)	FRAP (μmol/g)	TAC (mg CGE/100 g)	Sucrose (%)	Glucose (%)	Fructose (%)
Talesh	0.76b	18.63b	0.58a	31.79b	3.83a	84.84b	93.18b	60.84b	9.38b	3.27b	0.76b	1.30ab	0.94b
Langarud	0.63b	20.96a	0.55a	38.12a	3.75b	94.19a	104.91a	67.12a	10.68a	3.49a	0.79a	1.32a	0.98a
Rasht	1.20a	17.40c	0.63a	27.50b	3.71b	74.07c	88.37c	57.25c	8.48c	2.64c	0.74b	1.38b	0.91c
Significance levels (p)	**	**	n.s	**	**	**	**	**	**	**	**	^*^	**

n.s. Indicates no-significant and significant differences

^*^ and ^**^ Indicate significant differences at *p* < 0.05 and 0.01, respectively

Mean values with different letters within the same column are significantly different (*p* < 0.05)

The polyphenolic profile of kiwifruit is greatly influenced by the environment, including the amount of sunlight, temperature, rainfall, and nutritional factors [64]. Our results show that lower temperatures (17.32 °C and 13.24 °C, respectively) and higher precipitation (104.91 mm and 239.7 mm, respectively) in September and October resulted in higher TP in kiwifruit from Langarud (east of Guilan). These findings support those revealed by Mditshwa et al. [64] who reported that cool weather increases TP in pomegranate juice. Our results showed that the growing region was the main factor influencing the antioxidant compounds and nutritional quality of red-fleshed kiwifruit. The high TP, vitamin C, and anthocyanin contents of kiwifruit from Langarud may be responsible for its high antioxidant activity. Fruits produced in Langarud, which has a higher altitude and cooler summer temperatures, showed higher vitamin C, TP, and antioxidant activity than fruits from the other two regions at both ripening stages.

### Results of free sugars content (fructose, glucose, and sucrose)

The HPLC-ELSD method was used to analyze fructose, glucose, and sucrose in red kiwifruit. All three sugars were successfully identified in 25 min. Table 3 shows the sugar content of the kiwifruit samples examined in this study. Glucose and fructose were the predominant sugars in all samples, with sucrose content being lower. The fruits from Langarud (east of Guilan) had the highest fructose (0.98%), glucose (1.32%), and sucrose (0.79%), while the fruits from Rasht had the lowest contents. By the ripening of kiwifruit, the starch content decreases from 6% to near 0%, and the concentrations of total sugars and soluble solids increase up to 15% and 16%, respectively. Like other cellular components, sugar accumulation varies depending on cultivar, maturity, and environmental conditions. Increased variations between day and night temperature and increased UV radiations in higher altitudes, presents a complex of environmental parameters that leads to the increase in SSC and sugar content [65].

### Results of E-nose

**Fig. 7:**
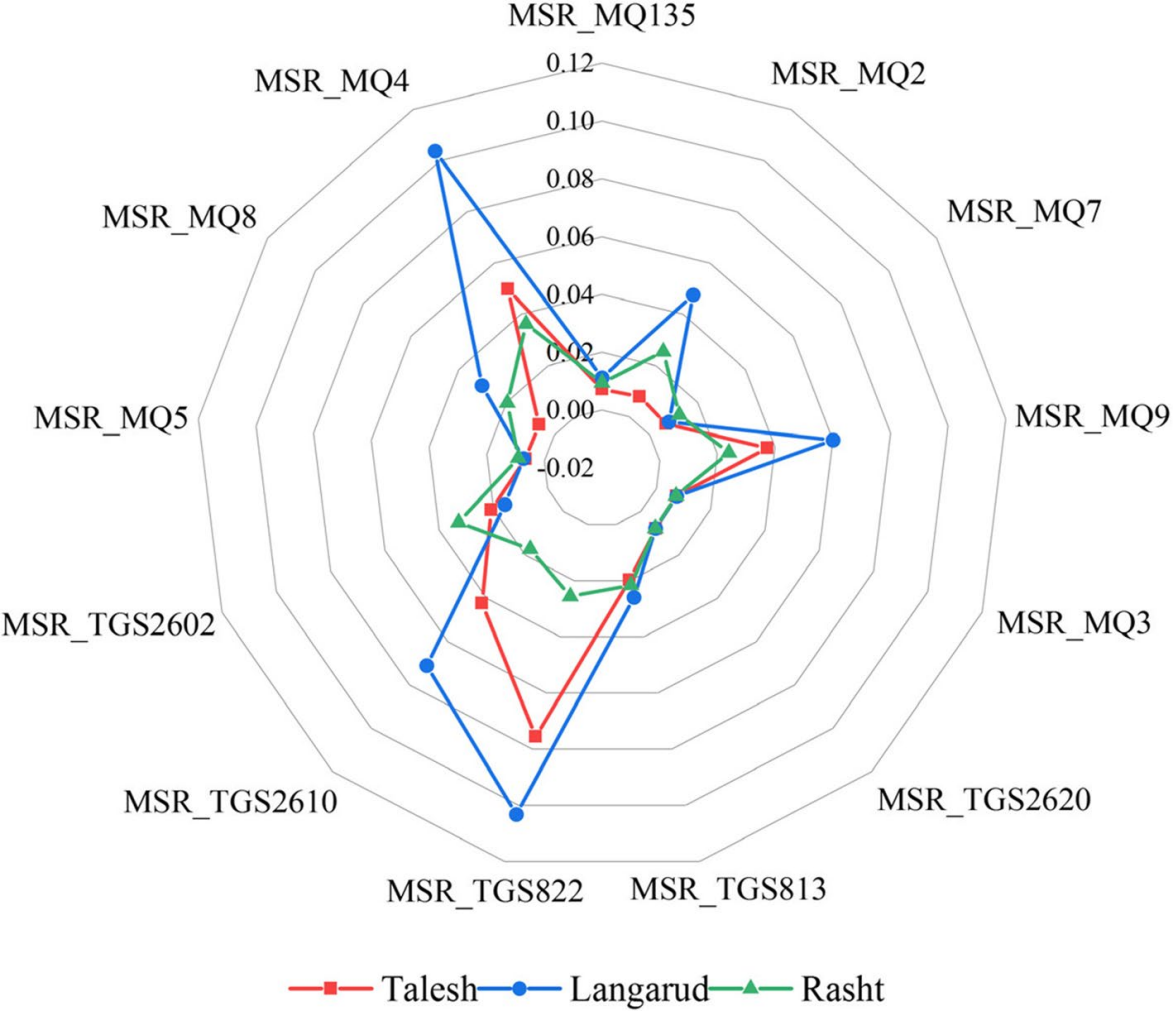
Radar plot of the MSR averages of e-nose sensors for red-fleshed kiwifruits of different growing region

Figure 7 shows the radar plots of the average e-nose MSR feature of different MQ sensors for three different growing geographical regions of ʻKhoniʼ kiwifruit. The graphs show that the maximum response of most sensors to the kiwifruit samples from the Langarud region is higher than the other two regions. This can be attributed to the higher concentration of volatile organic compounds in kiwifruit samples from Langarud region. Additionally, the differences between the average MSR values in most E-nose sensors are such distinct that it can be hoped to discriminate the samples of different origins based on e-nose data with a high accuracy.

**Fig. 8:**
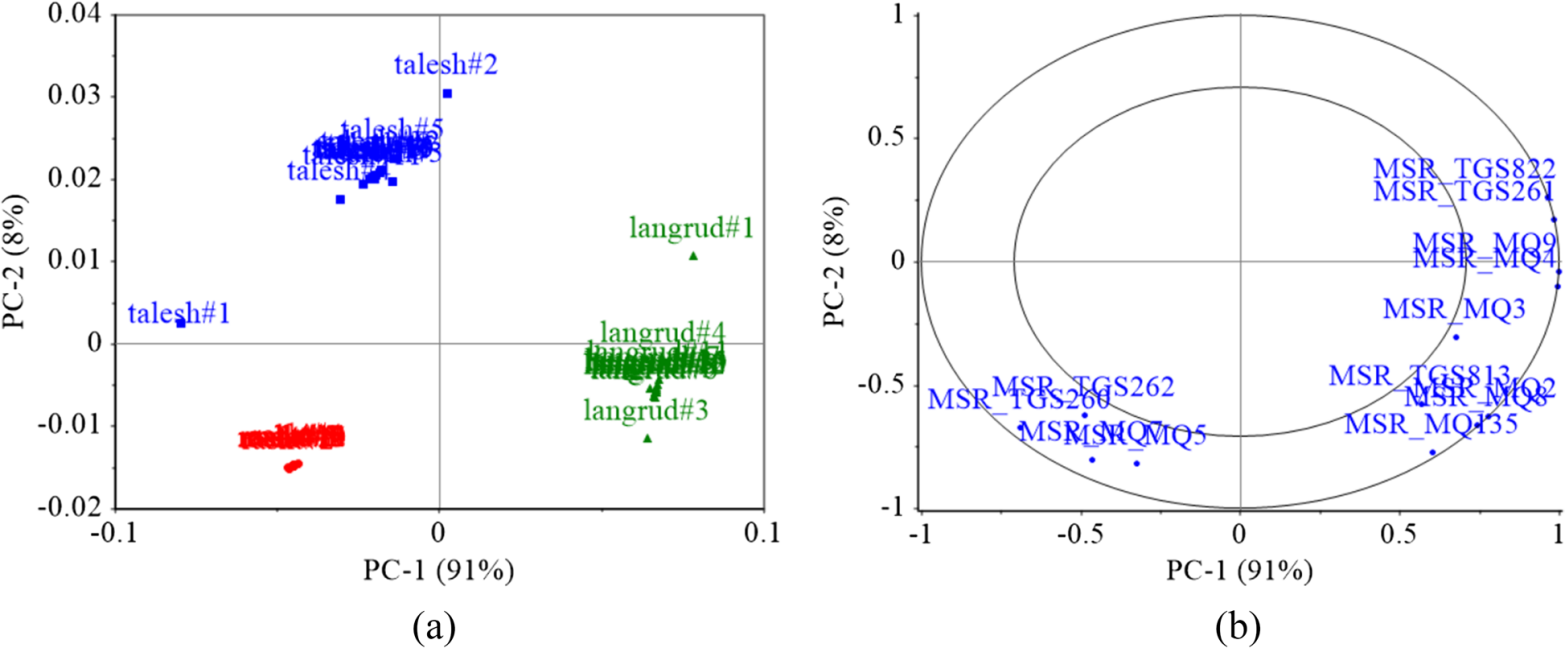
PCA score (**a**) and loading (**b**) plots for growing region discrimination of kiwifruit using e-nose data

The score plot of PCA analysis is presented in Fig. 8a. The first two principal components (PCs) extracted from the e-nose data accounted for 99% of the variance in the original e-nose data (PC1 = 91%, PC2 = 8%). Additionally, using these two PCs, samples from the three regions were completely discriminated. This demonstrates the high capability of the e-nose system to detect the growing location of red-fleshed kiwifruit based on the aroma-extracted features. The loading plot of the PCA is shown in Fig. 8b. The inner and outer circles represent 70% and 100% of the variance covered, respectively. The features closer to the boundary of the larger circle are more important for the detection and discrimination of the samples [66]. Figure 8b shows that most of the e-nose sensors have a significant role in the classification of fruits of different growing regions. Moreover, the MQ2, MQ4, MQ8, MQ9, MQ135, TGS822, and TGS2610 had the highest contributions.

Assessment of various PCA-SVM models for the classification of kiwifruits using e-nose data is presented in Table 4. It was found that the E-nose-based PCA-SVM classifier could successfully classify 'Khoni' kiwifruit samples based on their growing locations with an accuracy of 96.67% and an RMSE of 0.0201. This model discriminated between different origin classes with an accuracy of 93.33%, with only one case misclassified out of 15 test samples. These results demonstrate the high capability of the e-nose and PCA-SVM to identify the growing location of kiwifruit based on aroma characteristics.

**Table 4:** Performance criteria of the PCA-SVM model for classifying the kiwifruit growing region using e-nose, image-extracted, and fused data

Data	Optimal parameters	Calibration		Test	
		RMSE	Accuracy (%)	RMSE	Accuracy (%)
E-nose features	c = 1, $\gamma$=1	0.0201	96.67	0.0250	93.33
Image features	c = 100, $\gamma$=10	0.0233	93.33	0.0253	93.33
Fused data	c = 10, $\gamma$=1	0.0141	100	0.0166	100

Table 5 presents the performance statistics of the most accurate PCA-SVR models for predicting the physicochemical attributes of kiwifruit based on e-nose features. This table shows that the e-nose-based models accurately predicted the kiwifruit quality characteristics.

**Table 5:** Performance criteria of the PCA-SVR models for predicting the kiwifruit physicochemical indices using e-nose data

Quality index	Optimal parameters	Calibration		Validation		Test	
		RMSE	R^2^ (%)	RMSE	R^2^ (%)	RMSE	R^2^ (%)
Firmness (kg/cm^2^)	c = 10, $\gamma$=10	0.0247	99.06	0.0532	95.21	0.0687	93.09
SSC (°Brix)	c = 10, $\gamma$=0.1	0.2467	98.66	0.4549	95.98	0.6750	93.15
TA (%)	c = 100, $\gamma$=1	0.0072	98.76	0.0187	87.85	0.0157	90.86
BAR	c = 10, $\gamma$=1	0.5918	98.57	1.3052	86.46	1.3855	85.10
pH	c = 10, $\gamma$=10	0.0088	99.34	0.0139	96.85	0.0161	95.66
Vitamin C (mg/100 g)	c = 10, $\gamma$=10	1.2344	99.08	1.8094	97.04	2.0064	96.34
TP (mg GAE/100 g)	c = 0.01, $\gamma$=10	0.8569	99.34	1.2964	97.46	1.5189	96.51
TAC (mg CGE/100 g)	c = 10, $\gamma$=10	0.0448	99.34	0.0669	97.41	0.0713	97.06
DPPH (%)	c = 10, $\gamma$=10	0.7743	99.08	1.1722	96.48	1.5315	94.13
FRAP (μmol/g)	c = 10, $\gamma$=10	0.1083	99.29	0.1875	96.28	0.2479	93.97

The R^2^ coefficients obtained by the E-nose-PCA-SVR model in the cross-validation stage ranged from 86.46% for predicting kiwifruit BAR to 97.46% for predicting pH. This demonstrates the high capability of e-nose data in predicting the quality characteristics of kiwifruits from different orchard regions. In a previous study, the highest correlation coefficient (R) values between the reference and e-nose-based predicted SSC, firmness, and pH of pears were 0.93, 9.3, and 0.54, respectively [67]. Du et al. [68] reported the successful prediction of firmness, SSC, TA, and BAR measures of red kiwifruits using e-nose features and the PCA-SVR model. Accurate prediction (R^2^ > 92%) of firmness, pH, and SSC measures of cucumber was also obtained by Feng et al. [69] using the PCA-SVR model and e-nose-extracted variables.

### Results of CVS

**Fig. 9:**
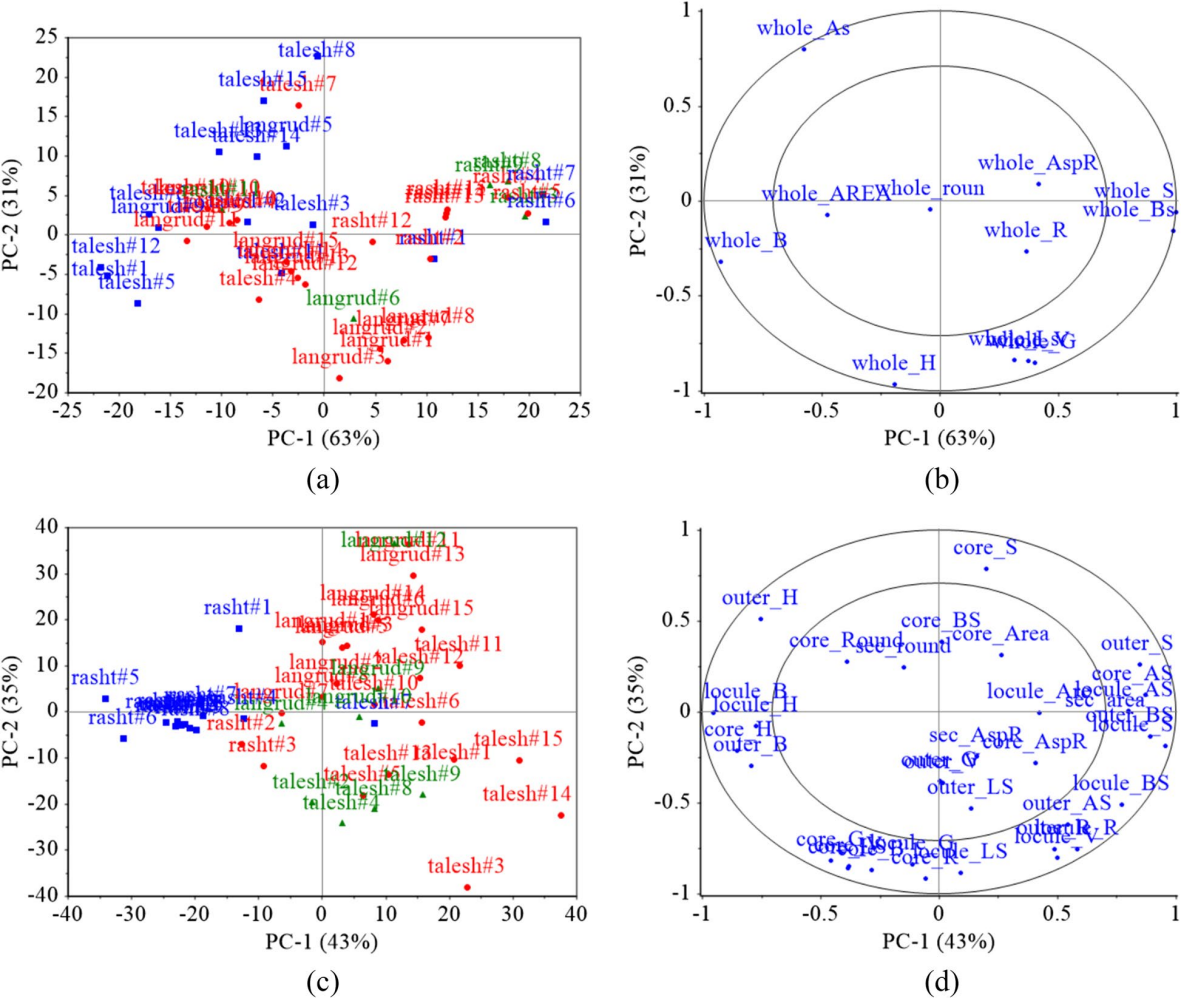
PCA score (**a**, **c**) and loading (**b**, **d**) plots for growing region discrimination of kiwifruit using image-extracted features from whole fruits (**a**, **b**) and middle-cult fruits (**c**, **d**)

Regarding the data extracted from the CVS, the PCA analysis was performed on the visual features of whole kiwifruits and those data obtained from fruit middle-cut images. Figure 9a shows the score plot of the first two principal components (PCs) constructed from the whole fruit image-extracted data. Although the first two PCs accounted for 94% of the variance of the image-extracted data (PC1 = 63% and PC2 = 31%), there is no appropriate discrimination between different locational classes of fruit. Considering the high coverage of the data variance by the first two PCs and the lack of proper separation of the samples (Fig. 9a), it can be concluded that the data extracted from the appearance of whole fruits does not have a suitable ability to identify the kiwifruit growing region. Additionally, Fig. 9b shows that the color components extracted from the surface of whole kiwifruits of different locations are more different than their shape characteristics.

Figure 9c shows the PCA score plot of the kiwifruit middle-cut image features. Although the first two PCs in this case covered only 78% of the data variance (PC1 = 43% and PC2 = 35%), the separation of different kiwifruit categories is better than that achieved by whole fruit image features. Additionally, by investigating other components, it was observed that incorporating the third (PC3 = 10%) and fourth (PC4 = 5%) components increases this variance coverage up to 93%. Figure 9d shows that the color features have a greater contribution than the shape features, and in particular, the color values of the locule segment were more significant. Therefore, it can be concluded that the difference in the place of kiwifruit growth has the greatest effect on the color characteristics of the fruit locule.

The first four PCA vectors were generated from the data extracted from the images of whole and middle-cut kiwifruits. These PC vectors were used to distinguish the growing location of kiwifruit samples. The statistics of the most accurate PCA-SVM model are reported in Table 4. As shown in Table 4, the image-based PCA-SVM model was less accurate in the calibration stage than the classifier developed using e-nose data. However, the developed model classified the kiwifruits from different regions with an accuracy similar to that of the e-nose-based PCA-SVM (93.33%). This demonstrates that the combination of appearance characteristics extracted from kiwifruits' whole and middle-cut images provides useful information for identifying the fruit growth location.

The results of predicting kiwifruit quality indicators using appearance characteristics are shown in Table 6. Vision-based models were less accurate than e-nose-based models. However, according to model validation measures, although the performance of the vision-SVR model in predicting some important features such as BAR (R^2^ = 68.36%) and pH (R^2^ = 78.15%) was inadequate, it was able to predict parameters such as firmness, TAC, TP, vitamin C, and anthocyanin measures with high accuracy (R^2^ > 93%).

**Table 6:** Performance criteria of the PCA-SVR models for predicting the kiwifruit physicochemical indices using image-extracted data

Quality index	Optimal parameters	Calibration		Validation		Test	
		RMSE	R^2^ (%)	RMSE	R^2^ (%)	RMSE	R^2^ (%)
Firmness (kg/cm^2^)	c = 10, ${\varvec{\gamma}}$=1	0.0392	97.38	0.0630	93.25	0.0671	92.61
SSC (°Brix)	c = 100, ${\varvec{\gamma}}$=10	0.3531	97.30	0.7282	88.78	0.6133	91.40
TA (%)	c = 100, ${\varvec{\gamma}}$=10	0.0132	95.16	0.0210	84.76	0.0206	85.63
BAR	c = 10, ${\varvec{\gamma}}$=1	0.5952	97.50	1.9928	68.36	2.2889	62.18
pH	c = 10, ${\varvec{\gamma}}$=0.1	0.0223	91.84	0.0364	78.15	0.0360	78.70
Vitamin C (mg/100 g)	c = 100, ${\varvec{\gamma}}$=10	1.2718	98.99	1.8705	96.00	1.7641	96.53
TP (mg GAE/100 g)	c = 100, ${\varvec{\gamma}}$=10	0.8659	99.05	1.8235	93.92	2.2468	91.65
TAC (mg CGE/100 g)	c = 10, ${\varvec{\gamma}}$=10	0.0456	99.20	0.0671	97.36	0.0774	96.52
DPPH (%)	c = 100, ${\varvec{\gamma}}$=10	0.7993	99.02	1.1814	96.45	1.4962	94.33
FRAP (μmol/g)	c = 100, ${\varvec{\gamma}}$=10	0.1093	99.13	0.2180	94.69	0.2841	92.01

It was reported by Li et al. [70] that the shelf life storage time, SSC, firmness, and TP values of Hayward kiwifruit are highly correlated (absolute R > 0.92) to RGB color values. In a recent study, image processing was used by Fashi et al. [71] for the accurate classification of pomegranate fruit based on pH value using artificial intelligence.

### Results of data fusion strategy

The fusion of olfactory and visual sensors data was used to classify kiwifruit samples based on their growing region and to estimate the fruit's quality characteristics. The results are as follows:

As shown in Table 4, the data fusion-based PCA-SVM classifier with a penalty value of 100 and a gamma value $(\gamma )$ of 10 achieved the best classification performance for kiwifruit geographical origin, with the smallest RMSE (0.0141) and classification accuracy of 100%. Evaluation of this model on test data resulted in an RMSE of 0.0163. The classification accuracy of the PCA-SVM model in test stage was also obtained to be 100%. This demonstrates the advantage of data fusion for improving classification model performance, as it was able to completely separate the red-fleshed kiwifruit samples from different regions.

Wu et al. [48] reported that fusing E-nose and e-tongue data can improve the accuracy of discriminating apple fruits based on geographical origin and variety, compared to using either system individually. The accuracy of the PCA-SVM classifier for classifying apple fruits using combined data reached 100%.

Table 7 presents the results of data fusion-based PCA-SVR models for predicting the physiochemical parameters of kiwifruit. A comparison of Table 7 with the performance data of Tables 5 and 6 reveals that the data fusion-based PCA-SVR models had the highest accuracy compared to the individual vision and olfactory datasets. The data fusion resulted in validation R^2^ values ranging from 90.17% for BAR prediction to 98.57% for pH prediction. These models were subsequently evaluated using separate data and high performance values were obtained according to Table 7. These high performance values show the high capability of combined systems based on E-nose and CVS technologies in reliably predicting the physiochemical parameters of red-fleshed kiwifruit.

**Table 7:** Performance criteria of the PCA-SVR models for predicting the kiwifruit physicochemical indices using the fusion of e-nose and image-extracted data

Quality index	Optimal parameters	Calibration		Validation		Test	
		RMSE	R^2^ (%)	RMSE	R^2^ (%)	RMSE	R^2^ (%)
Firmness (kg/cm^2^)	c = 10, $\gamma$=10	0.0212	99.55	0.0471	96.35	0.0621	94.54
SSC (°Brix)	c = 10, $\gamma$=10	0.2031	99.14	0.4236	96.10	0.4168	96.19
TA (%)	c = 100, $\gamma$=1	0.0050	99.14	0.0143	92.70	0.0115	94.76
BAR	c = 10, $\gamma$=10	0.5864	98.90	1.1404	90.17	0.9751	92.78
pH	c = 10, $\gamma$=1	0.0075	99.58	0.0103	98.57	0.0181	95.90
Vitamin C (mg/100 g)	c = 1, $\gamma$=1	1.1618	99.53	1.7298	97.11	1.7317	97.10
TP (mg GAE/100 g)	c = 100, $\gamma$=1	0.8566	99.36	1.2053	97.81	1.2460	97.63
TAC (mg CGE/100 g)	c = 100, $\gamma$=10	0.0415	99.82	0.0662	97.51	0.0671	97.40
DPPH (%)	c = 10, $\gamma$=1	0.7554	99.56	1.1438	96.95	1.1539	96.88
FRAP (μmol/g)	c = 100, $\gamma$=0.1	0.0893	99.37	0.1469	98.03	0.1545	97.86

**Fig. 10:**
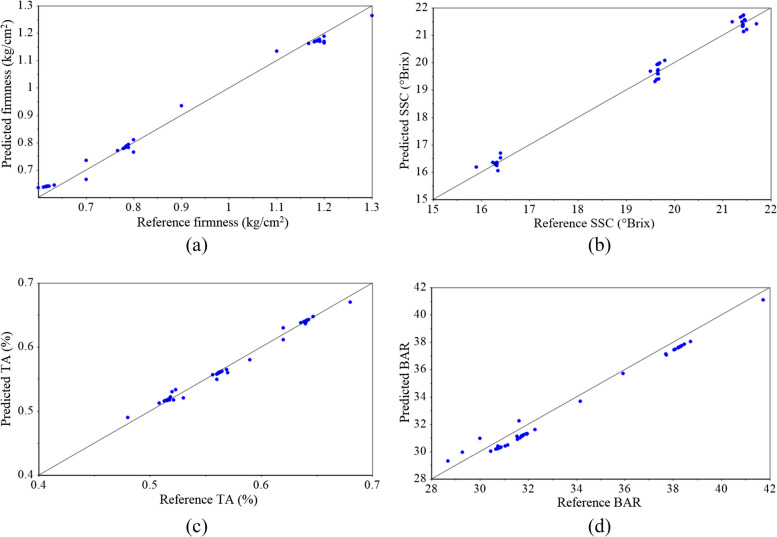
Results of data fusion-based PCA-SVR models for prediction of kiwifruit firmness (**a**), SSC (**b**), TA (**c**), and BAR (**d**)

**Fig. 11:**
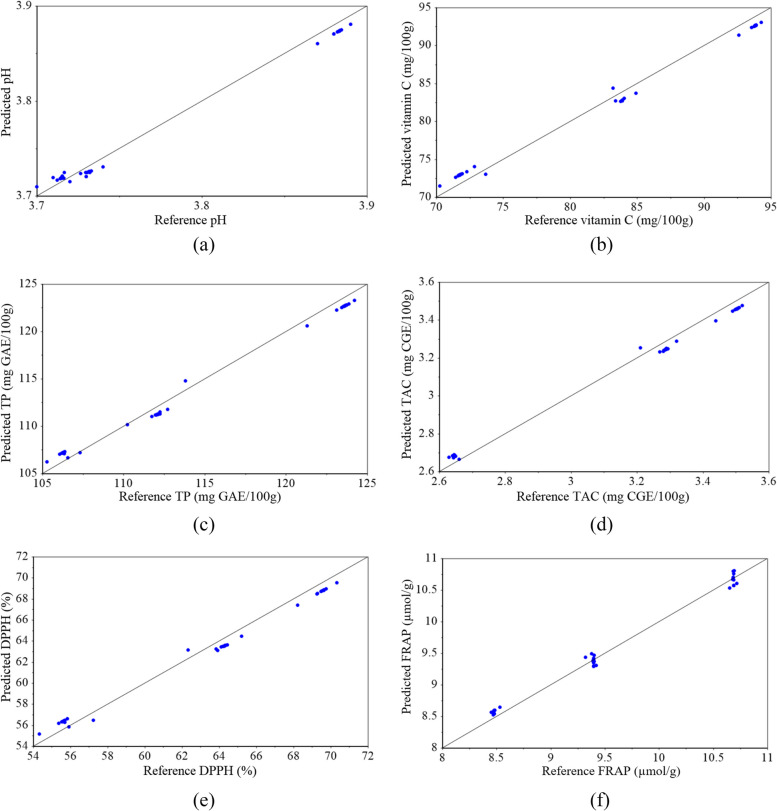
Results of data fusion-based PCA-SVR models for prediction of kiwifruit pH (**a**), vitamin C (**b**), TP (**c**), TAC (**d**), DPPH (**e**), and FRAP (**f**)

The graphical results of the data fusion-based PCA-SVR models are presented in Figs. 10 and 11. The proximity of the points to the target line (black diagonal line) shows the high accuracy of the PCA-SVR prediction models.

By referring to Tables 4, 5, 6 and 7, it can be seen that although for many attributes, the accuracies of the individual CVS and e-nose systems were acceptable, for some features such as BAR and TA. Additionally, the accuracies of both systems were low. For some other attributes (such as SSC and pH), the accuracy of the CVS system was not appropriate. Therefore, data fusion is important for increasing the prediction accuracy to an acceptable level. Furthermore, in the case of fruit origin classification, the use of combined data significantly increased the classification accuracy from 93 to 100%.

**Fig. 12:**
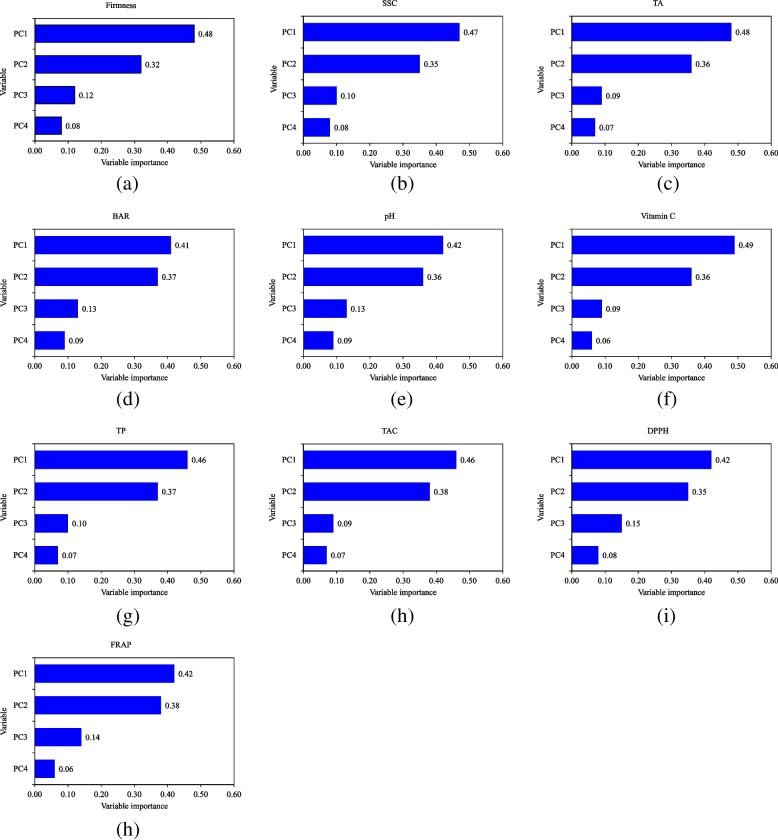
PC importance plots of PCA-SVR models for prediction of kiwifruit firmness (**a**), SSC (**b**), TA (**c**), BAR (**d**), pH (**e**), vitamin C (**f**), TP (**g**), TAC (**h**), DPPH (**i**), and FRAP (**j**)

Eventually, Fig. 12 represents the variable importance plots of SVR models for the prediction of quality attributes using the PC vectors derived from the fusion of CVS and e-nose data. It can be observed that the first and second PCs have the largest contribution in predicting the kiwifruit physiochemical attributes and are significantly more important than the other two PCs. The first PC, which covered the largest proportion of data variance, was more important than the second one. Moreover, the sum of the relative importance values of the third and fourth PCs also has a large proportion. This shows that the third and fourth PCs cannot be ignored since they have a meaningful effect in increasing the prediction performance of SVR models.

Although no studies have been found on the combination of electronic olfactory and vision systems for the detection of the geographical origin of crops, Huang et al. [53] reported that fusing CVS and e-nose information can result in more robust modeling of tomato ripeness classification and hardness prediction than using either appearance or aroma features alone. The combination of artificial nose and eye sensors also promoted the freshness classification performance of spinach rather than the separate systems [72].

The high accuracies achieved in this study demonstrate the high potential of the combined e-nose-e-vision system for detecting the growing region and predicting the quality attributes of kiwifruit. The electronic nose tests in this study were completely non-destructive. However, a limitation of this system is that the fruit must remain inside a closed container for a certain time to allow the space inside the container to be filled with the fruit's aroma. This increases the test time, which is a disadvantage for real-time analysis. In the case of CVS, although it is inherently capable of non-destructive and real-time evaluation, in this study, it was necessary to cut the fruit to obtain the differentiating information in the middle-cut section image, which caused destructive analysis. Besides, the accuracy of the individual CVS was insufficient for some physiochemical attributes.

Therefore, if a single, non-destructive recognition and prediction system is desired, the e-nose system is a better option. Additionally, we can explore ways to reduce the time the fruit must remain in the sample container. However, if destructiveness and time are not important, the proposed fused system is a good option for a more comprehensive and reliable system.

## Conclusion

The geographical growing region of horticultural fruits has an essential role in the quality of the harvested product. This study investigated the use of a combined e-nose and e-eye system to discriminate among red-fleshed kiwifruits from three different geographical regions in northern Iran. The following observations were made:

1. The fruit firmness, soluble solid content (SSC), Brix-acid ratio (BAR), pH, vitamin C, total phenolic content (TP), total anthocyanin content (TAC), antioxidant capacity, and free sugars of different regions were significantly (*p* < 0.01) different, but the titratable acidity (TA) of different fruits did not show a significant difference (*p* < 0.05).
2. The fusion of image-extracted and aroma-extracted features improved the accuracy of classification and prediction models compared to individual systems.
3. The data fusion-based PCA-SVM model achieved a 100% classification rate in detecting kiwifruits from different regions.
4. The data fusion-based PCA-SVR models achieved R^2^ values of 96.35%, 96.10%, 92.70, 90.17%, 98.57%, 97.11%, 97.81%, 97.51%, 96.95%, and 98.03% for predicting firmness, SSC, TA, BAR, pH, vitamin C, TP, TAC, DPPH, and FRAP, respectively.
5. The high accuracy measures obtained in this study demonstrate the high capability of the fusion of e-nose and e-eye systems for monitoring the quality attributes and discriminating among the growing regions of kiwifruit.
6. The results of this study provide valuable insights into the development of artificial sensing systems for the rapid and accurate regional, physical, and biological evaluation of kiwifruit.

## Data Availability

The experimental data supporting the findings of this research will be available on reasonable request.
